# Cross-sectional and prospective associations between sleep, screen time, active school travel, sports/exercise participation and physical activity in children and adolescents

**DOI:** 10.1186/s12889-018-5610-7

**Published:** 2018-06-07

**Authors:** Knut Eirik Dalene, Sigmund A. Anderssen, Lars Bo Andersen, Jostein Steene-Johannessen, Ulf Ekelund, Bjørge H. Hansen, Elin Kolle

**Affiliations:** 1 Department of Sports Medicine, the Norwegian School of Sport Sciences, Oslo, Norway; 2 Faculty of teacher Education and Sport, Western Norwegian University of Applied Sciences, Campus Sogndal, Sogndal, Norway; 3 Faculty of health sciences, Kristiania University College, Oslo, Norway

**Keywords:** Sleep, Screen time, Active travel, Sport, Exercise, Physical activty, Accelerometer

## Abstract

**Background:**

The aim of this study was to investigate how sleep, screen time, active school travel and sport and/or exercise participation associates with moderate-to-vigorous physical activity (MVPA) in nationally representative samples of Norwegian 9- and 15-y-olds, and whether these four behaviors at age nine predict change in MVPA from age nine to 15 years.

**Method:**

We pooled cross-sectional accelerometer and questionnaire data from 9- (*n* = 2366) and 15-y-olds (*n* = 1554) that participated in the first (2005/06) and second (2011/12) wave of the Physical Activity among Norwegian Children Study to investigate cross-sectional associations. To investigate prospective associations, we used data from a sub-sample that participated in both waves (at age nine and 15 years, *n* = 517).

**Results:**

Cross-sectional analyses indicated a modest, inverse association between screen time and MVPA among 9- (− 2.2 min/d (95% CI: -3.1, − 1.3)) and 15-y-olds (− 1.7 min/d (95% CI: -2.7, − 0.8)). Compared to their peers with 0–5 min/d of active travel to school, 9- and 15-y-olds with ≥16 min/d accumulated 7.2 (95% CI: 4.0, 10.4) and 9.0 (95% CI: 3.8, 14.1) more min/d of MVPA, respectively. Nine-y-old boys and 15-y-olds reporting ≥8 h/week of sports and/or exercise participation accumulated 14.7 (95% CI: 8.2, 21.3) and 17.9 (95% CI: 14.0, 21.8) more min/d of MVPA, respectively, than those reporting ≤2 h/week. We found no cross-sectional association between sleep duration and MVPA in either age group. None of the four behaviors predicted change in MVPA from age nine to 15 years (*p* ≥ 0.102).

**Conclusion:**

Active travel to school and sport/exercise participation may be important targets for future interventions aimed at increasing MVPA in children and adolescents. However, future studies are needed to determine causality.

**Electronic supplementary material:**

The online version of this article (10.1186/s12889-018-5610-7) contains supplementary material, which is available to authorized users.

## Background

Convincing evidence has emerged of a pronounced association between low levels of physical activity (PA) and an adverse metabolic profile already at a young age [1, 2]. Therefore, it has become a global priority to increase PA in children and adolescents [3]. Research conducted over the last two decades has identified a multitude of factors potentially important for the promotion of PA in children and adolescents [4]. This knowledge has aided development of interventions designed to increase young people’s PA, but unfortunately, many such interventions have only had limited or moderate success thus far [5–7]. Therefore, there is undeniably a continued need to increase our knowledge about modifiable factors potentially influencing PA in children and adolescents.

Some previous research has shown sleep duration [8, 9], screen time [10–13], active school travel [14–18], and sport/exercise participation [19, 20] to be associated with PA in children and adolescents. The observed associations between the two former behaviors and PA has recently led some authorities to include recommended levels of sleep and screen time to their PA guidelines for children [21]. However, the links between all these four potentially modifiable behaviors and PA stem predominantly from cross-sectional studies [16, 22–24], limiting causal inference.

Prospective studies examining determinants of PA have usually modelled these associations as change in the exposure with change in the outcome [20, 25–28], which does not determine the direction of association [29]. As an example, when an association between maintenance/adoption of organized sport participation associates with a beneficial change in PA, it is impossible to rule out that children who are more active and fit choose to continue or adopt organized sport participation [20]. Therefore, one cannot infer that organized sport participation predicts a higher PA level at a later time point.

In the Physical Activity among Norwegian Children Study (PANCS), we have collected data on PA, sleep duration, screen time, active school travel and sport/exercise participation in randomly selected, nationally representative samples of 9- and 15-y-olds in 2005–06 and 2011–12. In addition, a sub-sample of the participants were followed prospectively from age nine (2005–06) to 15 (2011–12) years. To inform future public health strategies and interventions for children and adolescents, we examined the cross-sectional and prospective associations between sleep duration, screen time, active school travel, sport/exercise participation, and PA.

## Methods

### Participants

PANCS is designed to monitor secular and longitudinal changes in PA in nationally representative samples of children and adolescents [30], and serves as the national PA surveillance system in Norway. The current study used pooled cross-sectional data from the first and second wave of PANCS (PANCS1 and PANCS2), and data collected from a sub-sample of participants followed prospectively from age ~ 9 years in PANCS1 to age ~ 15 years in PANCS2.

In PANCS1 (2005/06), we recruited 9- and 15-y-olds using a cluster sampling technique with schools as the primary unit. All fourth and tenth graders from schools that agreed to take part in the study were invited. In PANCS2 (2011/12), we recruited a new nationally representative sample of 9-y-olds using the same sampling technique as in PANCS1, whereas 15-y-olds were recruited either individually based on previous participation in PANCS1 (prospective sample) or from a random sample of lower secondary schools (cross-sectional sample).

### Anthropometrics

We measured height to 0.1 cm using a wall-mounted measuring tape, weight to 0.1 kg using Seca 770 and 877 scales (SECA GmbH, Hamburg, Germany) and calculated body mass index (BMI) using the standard formula (kg/m^2^). In PANCS1, the participants wore underwear during anthropometric measurements, whereas in PANCS2, they wore gym shorts and a t-shirt. Therefore, we subtracted 0.3 kg from the PANCS2 participants’ measured weight.

### Socioeconomic status

We categorized the participants into three socioeconomic status (SES) groups based on the parent with the highest education level. The parents self-reported this information in PANCS1, whereas Statistics Norway provided the information in PANCS2. Categories were coded “low”, (primary school or lower secondary school), “middle” (high school (vocational or general studies) and “high” (University College or University).

### Physical activity

We assessed PA using ActiGraph accelerometers (ActiGraph, LLC, Pensacola, Florida, USA). In PANCS1, we used the CSA 7164 model. In PANCS2, we used the GT1M and GT3X+ models. We used the RIU (K64, Computer Science & Application Inc., Shalimar, FL) and ActiLife software (ActiGraph, LLC, Pensacola, Florida, USA) to initialize and download the accelerometers PANCS1 and PANCS2, respectively, and KineSoft (v3.3.76; KineSoft, Loughborough, United Kingdom) for further processing of the accelerometer data.

We programmed the accelerometers to start recording at 06:00 on the day after the participants received them, and to sample activity counts in 10 s epochs. During school visits, we instructed the participants to wear the monitor on their right hip for four (PANCS1) and seven (PANCS2) consecutive days, and to remove the monitor for sleep and water based activities only. The different number of monitoring days is due to the limited storage capacity of the CSA 7164 model compared to the two newer models.

After exclusion of data recorded on weekend days, data recorded from 00:00–06:00 and intervals of ≥20 consecutive minutes with no activity counts recorded, we deemed all files with ≥2 weekdays consisting of ≥480 min of activity count recordings eligible for analysis.

In order to investigate average time spent in MVPA on weekdays, we applied a cut-point of ≥2000 counts∙min^− 1^ (CPM) scaled to match the 10 s epochs used, and divided all time spent in MVPA by the number of valid assessment days. This MVPA cut-point was developed for the European Youth Heart Study (EYHS) [31], is based on several validation studies and equivalent to a walking speed of > 4 km/h in children and adolescents [32].

### Sleep, screen time, active school travel and sport/exercise participation

A detailed description of the questions used to assess sleep duration, screen time, active school travel and sport/exercise participation is provided as online supporting information (Additional file 1: Table S1). The 9-y-old participants were assisted by their parents when answering the questionnaires, whereas the 15-y-olds answers were self-reported.

Participants reported when they usually got out of bed and went to bed on schooldays. To estimate sleep duration, we subtracted and added 0.5 h to the lower (“Before 06:30/20:00”) and upper (“After 08:00/24:00”) categories, respectively, and used the halfway point within remaining categories (e.g. “06:30–07:00” recoded 06:45). We then applied the following algorithm to approximate sleep duration on a numeric, continuous scale: *((24:00 – “bed time”) + (00:00 + “out of bed”)) = sleep duration*. This yielded 12 and 11 different sleep durations ranging from 5.75 to 12.25 h/night in 9- and 15-y-olds, respectively.

We computed screen time by combining information from three questions in the questionnaire. The participants indicated how many hours they usually watched TV before and after school, and how many hours they usually spent in front of a computer or with a videogame on schooldays. To approximate total screen time on a numeric scale, we used the midway point in the second lowest (“Less than 1 hour” recoded 0.5) to the second highest categories (e.g. “Between 3 and 4 hours” recoded 3.5) and added 0.5 h to the highest categories (e.g. “More than 4 hours” recoded 4.5). We then summed TV time before school, TV time after school and PC/videogame time on weekdays. This yielded 17 and 18 different screen times ranging from zero to 9.5 h/day in 9-y-olds and 15-y-olds, respectively.

Participants reported their usual travel mode and duration of travel to school. Because of the limited number of participants in each possible travel mode + duration category, and to create an easily interpretable, ordered scale, we stratified the data into three groups; 0) No active travel or < 5 min of active travel; 1) between 6 and 15 min of active travel, and; 2) ≥16 min of active travel.

Participants indicated how many hours per week outside of school they did sports or exercised. Because of the limited number of participants in each category, we combined the lowest two categories (0 h and 1–2 h), the middle two categories (3–4 h and 5–7 h), and the upper two categories (8–10 h and 11 h or more). The participants were then grouped accordingly.

### Analysis

We performed all statistical analyses using Stata 13.1 (StataCorp. 2013. Stata Statistical Software: TX: StataCorp LP.). Cross-sectional differences at baseline between the analytical sample and those lost to follow-up (prospective study sample) were analyzed using simple linear regression (continuous dependent variables), simple logistic regression, and simple ordered logistic regression (ordinal dependent variables).

Cross-sectional associations between MVPA (dependent variable) and the independent variables (sleep, screen time, school travel mode and sports/exercise) at age nine and 15 years were analyzed separately using linear regression. All four models were adjusted for accelerometer wear time, sex, BMI and SES. To account for the potential influence of seasonal changes on young people’s PA [33, 34], we also adjusted the models for minutes of daylight on each participants first day of accelerometer monitoring.

Prospective associations between changes in MVPA from baseline to follow-up and predictor variables (baseline sleep, baseline screen time, baseline school travel mode and baseline sports/exercise), were analyzed using linear regression adjusted for accelerometer wear time, baseline MVPA, sex, baseline BMI, baseline SES and change in minutes of daylight between baseline and follow-up.

Because of the aforementioned cluster sampling, we used Statas *xtreg, re* command (generalized least-square, random effects) with school declared (*xtset*) as the panel, and incorporated school as a cluster variable in both cross-sectional and prospective analyses using the *vce cluster* option to obtain robust variance estimations. In the prospective analyses, we used school at baseline as the cluster variable. We excluded participants with missing values for any of the variables in the statistical models through listwise deletion.

Since there were more than five sleep and screen time durations, reasonably large sample sizes and the sleep and screen time data were normally distributed, we chose to treat sleep and screen time as a continuous variable in all the analyses [35].

Lastly, we fitted interaction terms in initial cross-sectional and prospective analyses to assess whether sex modified associations. In analyses where the interaction term had a *p*-value less than 0.1, we stratified the analyses by sex to investigate to what extent sex was a modifier.

## Results

### Cross-sectional associations

In PANCS1, we invited 1470 9-y-olds and 1348 15-y-olds to participate, of which 1306 (89%) and 993 (74%) agreed to take part in the study. In PANCS2, we invited 1945 9-y-olds and 1759 15-y-olds, of which 1421 (73%) and 1106 (63%) agreed to participate. Combined, this yields participation rates of 80 and 68% for the 9- and 15-y-old study samples, respectively. A total of 2366 9-y-olds and 1554 15-y-olds provided ≥2 valid weekdays of accelerometer data. Table 1 displays descriptive characteristics of the analytical sample.

**Table 1:** Background characteristics of the cross-sectional and prospective study samples (mean (SD) unless otherwise specified)

	Cross-sectional samples				Prospective sample			
	9-y-olds	n	15-y-olds	n	Baseline	n	Follow-up	n
Girls	49.4%		51.0%		48.9%		48.9%	
Age (years)	9.6 (0.4)	2366	15.3 (0.6)	1554	9.6 (0.4)	517	15.2 (0.7)	517
Height (cm)	138.7 (6.6)	2342	169.6 (8.3)	1490	139.1 (6.4)	515	169.8 (8.4)	477
Weight (kg)	33.9 (6.8)	2343	60.7 (11.4)	1474	33.4 (6.3)	515	59.8 (10.5)	469
BMI (kg∙m^−2^)	17.5 (2.6)	2340	21.0 (3.3)	1473	17.2 (2.4)	515	20.7 (3.0)	469
SES								
Low	6.8%	153	5.9%	82	5.8%	29	5.8%	29
Middle	37.7%	843	38.8%	543	35.5%	179	35.5%	179
High	55.5%	1243	55.4%	776	58.7%	296	58.7%	296
Acc. WT (min/d)	804.5 (64.5)	2366	840.2 (89.5)	1554	817.4 (66.7)	517	824.5 (84.5)	517
MVPA (min/d)	92.1 (30.6)	2366	68.6 (26.3)	1554	98.2 (33.3)	517	69.5 (25.6)	517
Sleep (hrs/d)^a^	9.7 (0.8)	2102	8.1 (0.9)	1165	10.3 (0.6)	478	7.5 (0.7)	382
Screen time (hrs/d)	2.6 (1.3)	2081	3.9 (1.6)	1209	2.4 (1.3)	476	3.9 (1.6)	399
Active transport								
0–5 min/d	43.7%	926	52.3%	652	38.6%	188	48.9%	204
6–15 min/d	35.8%	757	36.1%	450	40.5%	197	36.7%	153
≥ 16 min/d	20.5%	434	11.6%	1247	20.9%	102	14.4%	60
Sports/exercise								
≤ 2 h/week	36.1%	762	30.2%	373	30.8%	150	28.6%	117
3–7 h/week	53.1%	1119	46.8%	577	56.1%	273	43.8%	179
≥ 8 h/week	10.8%	228	23.0%	284	13.1%	64	27.6%	113

*SES*, socioeconomic status; Acc. *WT*, accelerometer wear time; *MVPA*, moderate-to-vigorous physical activity; hrs/d, hours per day; min/d, minutes per day; hrs/week, hours per week. ^a^Possible range 7.5–13.0 h/night (PANCS1); 5.5–13.0 h/night (PANCS2/pooled)

**Fig. 1:**
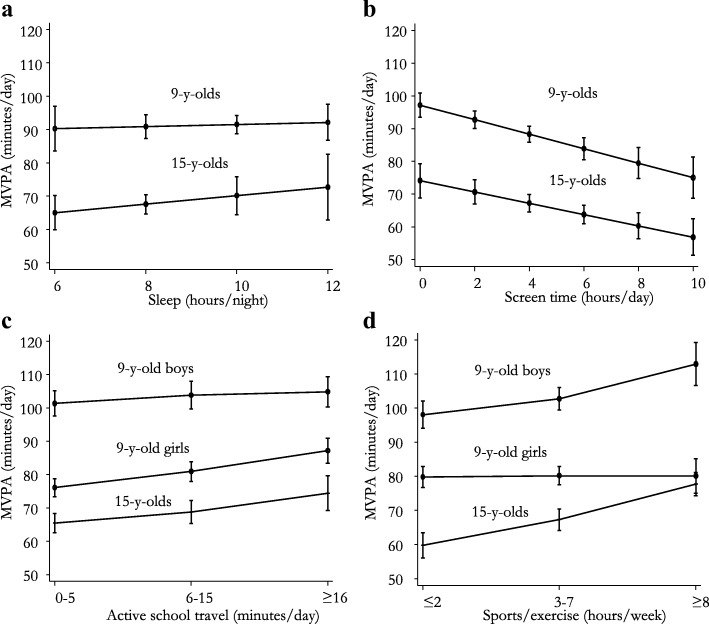
Cross-sectional associations between MVPA, sleep (**a**), screen-time (**b**), active school travel (**c**), sports/exercise participation (**d**). Mean values (95% CI) adjusted for accelerometer wear time, sex, body mass index (BMI), socioeconomic status (SES) and daylight. Nine-year-olds stratified by sex in C and D because of sex*active school travel (*p* = 0.006) and sex*sports/exercise (*p* < 0.001) interactions. MVPA, moderate-to-vigorous physical activity

Sleep duration was not associated with MVPA in either age group (Table 2, Fig. 1a). This association was unchanged in sensitivity analysis where we substituted the continuous sleep variable for a dichotomous variable based on suggested sleep recommendation attainment (9–11 h/night in 9-y-olds and 8–10 h/night in 15-y-olds, data not shown). 82.6 and 53.7% of 9- and 15-y-old participants reported sleeping the recommended minimum or more, respectively.

**Table 2:** Associations from cross-sectional analyses^a^

		9-y-olds			15-y-olds		
		MVPA (b (95% CI))	*p*	*n*	MVPA (b (95% CI))	*p*	*n*
Sleep		0.3 (−1.5, 2.2)	0.740	2053	1.3 (− 1.0, 3.6)	0.274	1120
Screen time		−2.2 (−3.1, −1.3)	< 0.001	2033	−1.7 (−2.7, −0.8)	< 0.001	1162
Active transport							
≤ 5 min/d		ref.		900	ref.		619
6–15 min/d		3.6 (0.9, 6.3)	0.009	742	3.3 (0.4, 6.2)	0.027	440
≥ 16 min/d		7.2 (4.0, 10.4) ^♀♂^	< 0.001	424	9.0 (3.8, 14.1)	0.001	137
Girls	≤5 min/d	ref.					
	6–15 min/d	4.6 (1.5, 7.8)	0.004				
	≥ 16 min/d	10.5 (6.8, 14.3)	< 0.001				
Boys	≤5 min/d	ref.					
	6–15 min/d	2.4 (−1.7, 6.6)	0.253				
	≥ 16 min/d	5.0 (0.4, 9.7)	0.033				
Sports/exercise							
≤ 2 h/week		ref.		736	ref.		350
3–7 h/week		2.2 (−0.1, 4.5)	0.062	1098	7.6 (4.3, 10.8)	< 0.001	555
≥ 8 h/week		9.2 (4.7, 13.7) ^♀♂^	< 0.001	225	17.9 (14.0, 21.8)	< 0.001	279
Girls	≤2 h/week	ref.					
	3–7 h/week	1.1 (−2.2, 4.4)	0.508				
	≥ 8 h/week	1.3 (−3.9, 6.5)	0.635				
Boys	≤2 h/week	ref.					
	3–7 h/week	4.5 (0.9, 8.2)	0.014				
	≥ 8 h/week	14.7 (8.2, 21.3)	< 0.001				

^a^Adjusted for accelerometer wear time, sex, BMI, SES and daylightMVPA, moderate-to-vigorous physical activity

b (95% CI), beta coefficient (95% confidence interval); min/d, minutes per day; hrs/week, hours per week; ♀♂, association modified by sex (*p* ≤ 0.036); ref., reference group

In 9- and 15-y-olds, we found inverse associations between screen time and MVPA (Fig. 1b), translating to 2.2 and 1.7 min/d less MVPA for each additional hour of screen time, respectively (Table 2). In sub-analyses, dichotomizing screen time based on suggested recommended levels revealed that 9-y-olds spending > 2 h/d in front of a screen accumulated 4.3 min/d (95% CI: 1.9, 6.8) less MVPA than 9-y-olds spending ≤2 h/d (*p* = 0.001). Among 15-y-olds, sex modified the association between the dichotomized screen time variable and MVPA (*p* = 0.014), and a difference between groups was only evident among boys (9.9 min/d (95% CI: 3.8, 16.1), *p* = 0.002). The proportions of 9- and 15-y-olds spending > 2 h/d in front of a screen were 53.5 and 81.3%, respectively.

In both 9- and 15-y-olds, active school travel was positively associated with MVPA (Table 2). Among 9-y-olds, sex modified the association (Fig. 1c), and when comparing those reporting 0–5 min/d and ≥ 16 min/d of active school travel, the differences were about twice as large in girls compared to boys (Table 2). Further, when comparing 9-y-olds reporting 0–5 min/d of active school travel to those with 6–15 min/d, the difference in MVPA was significant in girls, but not boys. Among 15-y-olds, the association between active school travel and MVPA was similar in girls and boys and appeared to be dose-dependent (Table 2, Fig. 1c).

Among 9-y-olds, sex modified the association between sport/exercise participation and MVPA (Fig. 1d, *p* < 0.001). Boys who reported doing ≥8 h/week of sports or exercise accumulated 14.7 min/d significantly more MVPA than boys reporting ≤2 h/week. No difference in MVPA was observed between girls in these two groups (Table 2). Sex-stratified analyses also revealed that boys, but not girls, in the 3–7 h/week group accumulated more MVPA than their peers in the ≤2 h/week group. Among 15-y-olds, both the 3–7 and ≥ 8 h/week groups accumulated significantly more MVPA than the ≤2 h/week group (Table 2). Sex did not modify these associations, and the associations appeared to be dose dependent (Fig. 1d).

### Prospective associations

In PANCS2, we were able to track and invite 1119 of the 1306 that participated in PANCS1 at age 9 years. Of these, 731 (65%) agreed to take part in PANCS2, of which 517 provided ≥2 valid weekdays of accelerometer data in both PANCS1 and PANCS2. Table 1 displays descriptive characteristics of the prospective study sample at baseline and follow-up.

Compared to those lost to follow-up, the prospective study sample had a lower BMI, slept more, reported less screen time and reported more time doing sports or exercising at baseline (online supporting information, Additional file 2: Table S2).

The mean (SD) interval between baseline and follow-up assessments was 5.6 (0.5) years, during which MVPA decreased by an average of almost 30 min/d (Table 1). Table 3 displays the results from the prospective analyses. Although the prospective associations between three of the four behaviors and MVPA go in the expected direction, sleep duration, screen time, active school travel and time spent doing sports or exercise at age 9 years were not significantly associated with change in MVPA from age 9 to 15 (Table 3). Dichotomizing sleep duration and screen time at baseline based on suggested recommendations did not change the results (data not shown).

**Table 3:** Associations from prospective analyses^a^

	MVPA (b (95% CI))^b^	*p*	*n*
Sleep	1.3 (−2.9, 5.5)	0.549	466
Screen time	−1.6 (− 3.5, 0.3)	0.102	464
Active transport			
≤ 5 min/d	ref.		186
6–15 min/d	2.2 (− 3.0, 7.4)	0.405	191
≥ 16 min/d	−2.0 (−9.2, 5.2)	0.580	98
Sports/exercise			
≤ 2 h/week	ref.		145
3–7 h/week	2.0 (−2.5, 6.6)	0.376	269
≥ 8 h/week	5.1 (−1.6, 11.8)	0.134	61

^a^Adjusted for accelerometer wear time, baseline MVPA, sex, baseline BMI, baseline SES and change in daylight from baseline to follow-up

^b^Beta values: impact of baseline sleep, screen time, active transport and sports/exercise on change in MVPA from baseline to follow-up

b (95% CI), beta coefficient (95% confidence interval); min/d, minutes per day; ref., reference group; MVPA, moderate-to-vigorous physical activity; hrs/week, hours per week

## Discussion

Our cross-sectional results suggested that screen time, active school travel and sports/exercise participation may influence habitual MVPA in both 9- and 15-y-olds. In contrast, we did not observe any association between these behaviors and objectively measured MVPA in prospective analyses.

### Sleep

Insufficient sleep is associated with several negative physical and mental health outcomes [22]. Thus, it is recommended that children (ages 6–13 years) and adolescents (ages 14–17 years) sleep 9–11 h/night and 8–10 h/night, respectively [36]. One hypothesis is that sufficient sleep facilitates a more physically active lifestyle, which has well-established health benefits in young people [37, 38]. If true, the associations between sleep and health outcomes might exist in synergy with associations between PA and health outcomes [39]. However, our results are in line with some [9, 25, 40–44] but not all [45, 46] previous studies and do not confirm the hypothesis that short sleep duration negatively affects MVPA. Further, in one of very few experimental studies investigating the effect of altering sleep duration on habitual MVPA [47], Hart et al. (2016) found no difference in objectively assessed MVPA between one week of decreased sleep (− 1.5 h./d) and one week of increased sleep (+ 1.5 h./d) in 8–11 year-old children [41].

### Screen time

The cross-sectional associations we observed corroborate a systematic review and meta-analysis conducted by Pearson et al. (2014) finding an overall small inverse association between screen time and PA [48]. Previous studies have also indicated that screen time during childhood is a poor predictor of objectively assessed PA [49]. In our cross-sectional study samples, every one-hour increase of screen time was only associated with ~ 2 min/d less MVPA. Thus, screen time and MVPA seems quite weakly associated, and our results do not indicate that Norwegian youth sacrifice a substantial amount of MVPA to engage in screen-based activities. Considering that a meta-analysis of 33 interventions aimed at reducing screen time in children and adolescents only showed a small overall effect [50], the potential of screen time reduction as a component in interventions aiming to increase MVPA seems limited. Nevertheless, studies are indicative of an indirect relationship between TV viewing and cardiovascular disease risk in young people [51–53]. Therefore, efforts made to limit TV viewing may have important public health implications, irrespective of its weak association with MVPA.

### Active school travel

Larouche et al. (2014) systematically reviewed 28 studies examining the association between active school travel and accelerometer assessed PA and found that the majority (*N* = 22) reported a positive association [16]. An interesting observation in our study is the stronger association in 9-y-old girls than in boys. Cooper et al. (2006) reported a similar observation in Danish 9-y-olds [54], but several studies have reported an association in boys only, including a study in Swedish and Estonian 9- and 15-y-olds [55]. This might indicate cultural differences, even between neighboring countries, and that facilitation and promotion of active school travel could be a valuable component in future interventions aiming to increase MVPA in young girls in Norway.

The seemingly dose-dependent relationship between active school travel and MVPA among 9-y-old girls and 15-y-olds observed in this study is similar to associations reported between active school travel distance and MVPA by others [56, 57]. Future studies separating walking and cycling as active behaviors are warranted to aid our understanding about the potential impact of these two behaviors during active transport on MVPA in young people and between genders.

Engaging with active travel during childhood may foster a positive attitude towards, and develop the skillset in, PA that is sustained throughout the life course. Therefore, we can hypothesize that active travel during childhood may convey self-efficacy regarding PA capacity, potentially lowering the perceived barriers towards PA later in life. However, our results do not indicate that active school travel during childhood is a predictor of MVPA during adolescents. Accelerometers have been shown to underestimate MVPA during cycling [58], and because the proportion of participants in the prospective study sample who cycled to school increased from 6.2 to 17.0% between baseline and follow-up, it is possible that adoption of a change of mode of transportation between age nine and 15 years may have masked a potential prospective associations in our study.

### Sports/exercise participation

Our results corroborate those from a systematic review suggesting a positive association between sport participation and MVPA [24]. We consider the strength of the associations we observed comparable to those reported by Hebert et al. (2015), which found leisure-time sport participation to associate with 5–20 min/d more MVPA, depending on the type of sport and frequency of participation [19]. However, we did not observe any association between sports/exercise participation and MVPA in 9-y-old girls. We can only speculate as to why girls and boys that report doing the same amount of sports/exercise have different levels of habitual MVPA. One possibility is that girls and boys accumulate different levels of MVPA during the same sports and/or exercise activities. There is however very few data available regarding activity levels of girls and boys during specific activities outside of school to support this. Although accelerometers do not have the ability to distinguish between PA types under free-living conditions, merging minute-by-minute data from accelerometers with activity logs can potentially facilitate investigation of gender differences in MVPA during specific activities in future studies. It is also possible that the 9-y-old girls and boys participated in different sports and/or exercise activities that yield different levels of MVPA.

A compensatory mechanism has been suggested when PA is high in one domain (e.g. during sport/exercise) [59], which might result in similar daily levels of MVPA among girls with different levels of sports/exercise participation. However, current research testing this “activity-stat” hypothesis is inconclusive, and does not suggest a sex difference [59].

Similar to active travel, we can hypothesize that participation in sports and exercise during childhood determines higher levels of PA later in life through an increased self-efficacy regarding PA capacity. Although there was a trend toward an association between sport/exercise participation at age 9 and change in MVPA from age 9 to 15, we cannot conclude that sports/exercise participation during childhood is a determinant of MVPA during adolescence in our study sample. This is supported by Brooke et al. (2014), who found no association between variety and frequency of sports and exercise activities at age 10 and MVPA at age 14, and Basterfield et al. (2014), who found no association between minutes per week of sports club participation at age 9 and MVPA at age 12 [60, 61]. Taken together, a general promotion of sports and/or exercise participation during childhood does not seem to protect against the well-established MVPA decline from childhood to adolescence [30]. However, future studies investigating the prospective association between specific types of sports and exercises and objectively assessed MVPA are warranted.

### Strengths and limitations

A major strength of this study is the large, population-based samples of children and adolescents and the high participation rates. Another strength is the objective measure of habitual MVPA, reducing the risk of biases associated with self-report [62]. Furthermore, 90% of the participants wore the monitor for an average > 720 min/d, indicating that the vast majority awake time was monitored. In addition, we adjusted the regression models for a number of covariates reducing the risk of confounding and we explored interactions with sex.

However, our results should be interpreted with the following limitations in mind. Although the attrition rate in the prospective study sample is comparable to similar studies, the differences detected in the lost to follow-up analyses indicated selection bias. Although generalizability is not required to detect associations, this makes it plausible that the results are not fully generalizable to a larger population of nine and 15-year old Norwegians. In addition, the relatively small prospective study sample does increase the risk of type II errors.

Further, the absolute validity of the questions used to measure the exposure variables is unknown. Although self- and proxy reported sleep show good correlation for questionnaires (*r* = 0.60–0.78) and diaries (*r* = 0.97) when compared with objective measures in children and adolescents, sleep times are usually overestimated [63]. Further, self-report measures provide reliable estimates of screen-time, yet their validity remains largely untested [64, 65]. Similarly, due a number of challenges, including logistical and privacy concerns, the absolute validity of self-reported, habitual active school travel and sport/exercise participation has not been quantified. Thus, even if other studies have used similar methods and we consider the face validity reasonable, this is a limitation. Another potential limitation is the use of inconsistent terminology when assessing weekday TV (before and after school) and computer/videogame use (weekdays) could have caused some of the participants to include educational computer use. Lastly, random measurement error is inherent when self-report is used to assess the quantity of behaviors in young people. This may lead to regression dilution bias [66], increasing the risk of type 2 errors.

Also of note is that we did not assess other aspects of the exposure variables that may be associated with MVPA. For example, we did not investigate whether sleep quality, different screen behaviors, different active travel modes to/from other destinations than school and participation in specific types of sport and exercise associates with MVPA.

Three of the four behaviors were specific to weekdays (Additional file 1: Table S1). Consequently, we also chose to exclude weekend MVPA to ease the interpretation of the findings. However, we cannot rule out that the behaviors are associated with weekend MVPA also. Since the question used to assess sport/exercise participation was not specific to weekdays (Additional file 1: Table S1), we reran the analysis using average daily MVPA for the full week as the dependent variable. Results from these analyses are presented as online supporting information (Tables S3a and S3b). Notably, the cross-sectional associations in Additional file 3: Table S3a are very similar to those presented in Table 2. However, it is also noteworthy that the trend toward a prospective association between sport/exercise participation at age 9 and change in MVPA from age 9 to 15 is stronger (Additional file 3: Table S3b).

Compositional data analysis is an emerging statistical approach to analyze associations between different behaviors and physical activity [67]. This was not possible in the current dataset, as we did not have access to 24 h/d movement data, but its application in future studies may offer additional insight.

Lastly, hip-worn accelerometers under-estimate non-ambulatory PA such as cycling [58], which will likely attenuate associations between active school travel and MVPA. The same is also probable for associations between the three other behaviors and MVPA in participants who were avid cyclists.

## Conclusion

In conclusion, this study adds to the growing body of evidence linking active school travel and participation in sport and exercise to habitual MVPA. In Norwegian children and adolescents, MVPA on weekdays does not however seem associated with sleep duration, and only weakly associated with screen time. Although we did not observe any prospective associations between any of the four behaviors investigated and MVPA, we believe our cross-sectional findings should encourage more studies to investigate whether altering active school travel and participation in sports and/or exercise impacts habitual MVPA using a randomized study design. Given the highly complex nature of the decline in MVPA from childhood to adolescence, we also encourage future observational studies to investigate prospective associations between additional aspects of these behaviors and MVPA.

## Additional files

Additional file1: **Table S1.** Questions and possible answers from the questionnaire used to asses sleep duration, screen time, active school travel and sports/exercise participation.) (DOCX 16 kb)

Additional file 2: **Table S2.** Results from loss to follow-up analyses. (DOCX 17 kb)

Additional file 3: **Table S3.** a Associations from cross-sectional analyses of the association between sport/exercise participation, minutes per day of MVPA on weekdays (mon-fri) and weekly (mon-sun) minutes per day of MVPA b: Associations from prospective analyses of the association between sport/exercise participation at age 9, minutes per day of MVPA on weekdays (mon-fri) at age 15 and weekly (mon-sun) minutes per day of MVPA at age 15^1^. (DOCX 14 kb)

## Abbreviations

BMI: Body mass index
MVPA: Moderate-to-vigorous physical activity
PA: Physical activity
PANCS: the Physical Activity among Norwegian Children Study
SES: Socioeconomic status
6−/9−/15-y-old(s): 6−/9−/15-year-old(s)
