# Supplementary material for: Cross-sectional and prospective associations between sleep, screen time, active school travel, sports/exercise participation and physical activity in children and adolescents
Source: BMC Public Health. 2018 Jun 7;18:705. doi: 10.1186/s12889-018-5610-7 (PMC5992852; doi:10.1186/s12889-018-5610-7)
Supplement: Supplementary file 1 — Table S1. Questions and possible answers from the questionnaire used to asses sleep duration, screen time, active school travel and sports/exercise participation.) (DOCX 16 kb) [file 12889_2018_5610_MOESM1_ESM.docx]

Table S1: Questions and possible answers from the questionnaire used to asses sleep duration, screen time, active school travel and sports/exercise participation)

| **Sleep duration** |  |
| --- | --- |
| When do you usually get out of bed on schooldays? | □ Before 06:30, □ Between 06:30 and 07:00, □ Between 07:00 and 07:30, □ Between 07:30 and 08:00, □ After 08:00^1^ |
| When do you usually go to bed on schooldays? | □ Before 20:00, □ Between 20:00 and 21:00, □ Between 21:00 and 22:00, □ Between 22:00 and 23:00, □ Between 23:00 and 24:00, □ After 24:00^2^ |
| **Screen time** |  |
| How many hours do you usually watch TV before school? | □ None, □ Less than 1 hour, □ Between 1 and 2 hours, □ More than 2 hours |
| How many hours do you usually watch TV after school? | □ None, □ Less than 1 hour, □ Between 1 and 2 hours, □ Between 2 and 3 hours, □ Between 3 and 4 hours, □ More than 4 hours^3^ |
| How many hours do you usually spend on a PC (to play games or surf the internet) or with a videogame (PlayStation, X-box or similar) on a weekday? | □ None, □ Less than 1 hour, □ Between 1 and 2 hours, □ Between 2 and 3 hours, □ Between 3 and 4 hours, □ More than 4 hours^4^ |
| **Active transport** |  |
| How do you usually get to school this time of year?^5^ | □ By car or motorcycle, □ By bus, tram, metro or train, □ Cycle, □ Walk |
| How long does it usually take you to get to school? | □ Less than 5 minutes, □ 6-15 minutes □ 16 to 30 minutes, □ 31 minutes to 1 hour, □ More than 1 hour |
| **Sports/exercise** |  |
| Outside of school: How many hours per week do you do sports/exercise that makes you breathe hard or sweat? | □ 0 hours, □ 1-2 hours, □ 3-4 hours, □ 5-7 hours, □ 8-10 hours, □ 11 hours or more |

^1^ In PANCS1, the highest category was "after 07:30"
^2^ In PANCS1, the highest category was "after 22:00"
^3^ In PANCS1, the highest category was "more than 3 hours"
^4^ In PANCS1, the highest category was "more than 3 hours"
^5^ "this time of year" was not specified in PANCS1
