# Supplementary material for: Cross-sectional and prospective associations between sleep, screen time, active school travel, sports/exercise participation and physical activity in children and adolescents
Source: BMC Public Health. 2018 Jun 7;18:705. doi: 10.1186/s12889-018-5610-7 (PMC5992852; doi:10.1186/s12889-018-5610-7)
Supplement: Supplementary file 2 — Table S2. Results from loss to follow-up analyses. (DOCX 17 kb) [file 12889_2018_5610_MOESM2_ESM.docx]

Table S2: Results from loss to follow-up analyses

|  | | **Study sample** | **n** | **Lost to follow-up** | **n** |
| --- | --- | --- | --- | --- | --- |
| Age (years) | | 9.63 (0.38) | 517 | 9.61 (0.39) | 785 |
| BMI (kg∙m^-2^) | | 17.17 (2.40)* | 515 | 17.55 (2.70) | 768 |
| Overweight (%)^2^ | | 14.17* | 515 | 20.83 | 768 |
| Obese (%)^2^ | | 2.72* | 515 | 4.95 | 768 |
| SES (%) | |  | 474 |  | 690 |
|  | Low | 6.54 |  | 8.55 |  |
|  | Middle | 38.40 |  | 37.83 |  |
|  | High | 55.06 |  | 53.62 |  |
| MVPA (min/d) | | 97.79 (34.08) | 517 | 95.29 (34.79) | 651 |
| Sleep (hrs./d) | | 10.33 (0.56)* | 478 | 10.25 (0.60) | 704 |
| Screen time (hrs./d) | | 2.43 (1.33)* | 476 | 2.67 (1.45) | 696 |
|  | TV time (hrs./d) | 1.49 (0.88)* | 479 | 1.61 (0.99) | 698 |
|  | Computer time (hrs./d) | 0.93 (0.80)* | 480 | 1.07 (0.87) | 706 |
| Active transport (%) | |  | 487 |  | 705 |
|  | 0-5 min/d | 38.60 |  | 41.56 |  |
|  | 6-15 min/d | 40.45 |  | 38.16 |  |
|  | ≥16 min/d | 20.94 |  | 20.28 |  |
| Sports/training (%) | |  | 487 |  | 698 |
|  | ≤2 hrs./week | 30.80 |  | 37.68 |  |
|  | 3-7 hrs./week | 56.06* |  | 50.72 |  |
|  | ≥8 hrs./week | 13.14* |  | 11.60 |  |

* Significantly different from those lost to follow-up (p≤0.050)
