# Supplementary material for: Cross-sectional and prospective associations between sleep, screen time, active school travel, sports/exercise participation and physical activity in children and adolescents
Source: BMC Public Health. 2018 Jun 7;18:705. doi: 10.1186/s12889-018-5610-7 (PMC5992852; doi:10.1186/s12889-018-5610-7)
Supplement: Supplementary file 3 — Table S3. a Associations from cross-sectional analyses of the association between sport/exercise participation, minutes per day of MVPA on weekdays (mon-fri) and weekly (mon-sun) minutes per day of MVPA b: Associations from prospective analyses of the association between sport/exercise participation at age 9, minutes per day of MVPA on weekdays (mon-fri) at age 15 and weekly (mon-sun) minutes per day of MVPA at age 151. (DOCX 14 kb) [file 12889_2018_5610_MOESM3_ESM.docx]

Table S3a: Associations from cross-sectional analyses of the association between sport/exercise participation, minutes per day of MVPA on weekdays (mon-fri) and weekly (mon-sun) minutes per day of MVPA ^1^

|  | | **9-y-olds (Girls / Boys ^♀♂^)** | | **15-y-olds** | |
| --- | --- | --- | --- | --- | --- |
|  | | **MVPA (b (95% CI))** | **n** | **MVPA (b (95% CI))** | **n** |
| Sports/exercise weekdays | |  |  |  |  |
|  | ≤2 hrs/week | ref. | 736 (437/299) | ref. | 350 |
|  | 3-7 hrs/week | 1.1 (-2.2, 4.4) / 4.5 (0.9, 8.2)* | 1098 (511/587) | 7.6 (4.3, 10.8)** | 555 |
|  | ≥8 hrs/week | 1.3 (-3.9, 6.5) / 14.7 (8.2, 21.3)** | 225 (73/152) | 17.9 (14.0, 21.8)** | 279 |
| Sports/exercise total week | |  |  |  |  |
|  | ≤2 hrs/week | ref. | 736 (437/299) | ref. | 350 |
|  | 3-7 hrs/week | 2.6 (-0.3, 5.4) / 4.8 (1.5, 8.0)** | 1098 (511/587) | 8.3 (5.2, 11.4)** | 555 |
|  | ≥8 hrs/week | 3.8 (-1.3, 9.0) / 15.9 (9.9, 21.8)** | 225 (73/152) | 20.0 (16.0, 24.1)** | 279 |

^1^Adjusted for accelerometer wear time, sex, BMI, SES and daylight
MVPA, moderate-to-vigorous physical activity; b (95% CI), beta coefficient (95% confidence interval); hrs/week, hours per week; ♀♂, association modified by sex (p≤0.036); *, p≤0.027; **, p≤0.009; ref, reference group

Table S3b: Associations from prospective analyses of the association between sport/exercise participation at age 9, minutes per day of MVPA on weekdays (mon-fri) at age 15 and weekly (mon-sun) minutes per day of MVPA at age 15^1^

|  | | **MVPA (b (95% CI)) ^2^** | **n** |  |
| --- | --- | --- | --- | --- |
| Sports/exercise weekdays | |  |  |  |
|  | ≤2 hrs/week | ref. | 145 |  |
|  | 3-7 hrs/week | 2.0 (-2.5, 6.6) | 269 |  |
|  | ≥8 hrs/week | 5.1 (-1.6, 11.8) | 61 |  |
| Sports/exercise total week | |  |  |  |
|  | ≤2 hrs/week | ref. | 145 |  |
|  | 3-7 hrs/week | 2.6 (-1.9, 7.1) | 269 |  |
|  | ≥8 hrs/week | 6.2 (-0.0, 12.5)^#^ | 61 |  |

^1^ Adjusted for accelerometer wear time, baseline MVPA, sex, baseline BMI, baseline SES and change in daylight from baseline to follow-up. ^2^ Beta values: impact of baseline sleep, screen time, active transport and sports/exercise on change in MVPA from baseline to follow-up.
b (95% CI), beta coefficient (95% confidence interval); ref., reference group; MVPA, moderate-to-vigorous physical activity; hrs/week, hours per week; ^#^ p=0.051.
